# Trans‐Tissue Effects of Hippocampus‐ and Blood‐Derived DNA Methylation Risk Scores on Bipolar Disorder Diagnosis

**DOI:** 10.1111/bdi.70078

**Published:** 2026-01-24

**Authors:** Kazutaka Ohi, Daisuke Fujikane, Kentaro Takai, Ayumi Kuramitsu, Yukimasa Muto, Shunsuke Sugiyama, Toshiki Shioiri

**Affiliations:** ^1^ Department of Psychiatry Gifu University Graduate School of Medicine Gifu Japan

**Keywords:** bipolar disorder, blood, diagnosis, hippocampus, methylation risk score

## Abstract

**Objectives:**

Bipolar disorder (BD) is a common psychiatric disorder with complex genetic and epigenetic underpinnings. This study aimed to investigate whether methylation risk scores (MRSs) derived from epigenome‐wide association studies (EWASs) for BD risk in living peripheral blood and postmortem hippocampal tissues are associated with BD diagnosis across tissues.

**Methods:**

DNA methylation data were analyzed from two datasets, including living peripheral blood samples (*n* = 40) and postmortem hippocampal tissues (*n* = 63) obtained from patients with BD and unaffected controls. Two EWASs using data from blood and hippocampal samples were performed to identify differentially methylated positions (DMPs), and MRSs for BD risk in blood and hippocampal samples were calculated by aggregating methylation effects across the genome. Associations between MRSs and BD diagnosis and the potential influences of genome‐wide significant (GWS) loci related to BD and health‐related confounding factors, such as smoking, body mass index (BMI), and suicide, on these associations were assessed.

**Results:**

Postmortem hippocampus‐derived MRSs for BD risk were significantly associated with BD diagnosis in blood samples (*R* ^2^ = 0.147, *p* = 0.026), whereas blood‐derived MRSs for BD risk showed no significant associations in postmortem hippocampal tissue. These findings were not primarily driven by CpG sites near GWS loci or health‐related confounders. However, when focusing specifically on a restricted subset of 49 CpG sites located near GWS loci, the MRSs were significantly associated with BD diagnosis (*R* ^2^ = 0.135, *p* = 0.030).

**Conclusions:**

Postmortem hippocampus‐derived MRSs may capture brain‐specific epigenetic changes associated with BD pathophysiology, reflecting their diagnostic relevance in living peripheral blood. Further studies with larger sample sizes and multitissue approaches are needed to validate these findings.

## 1 Introduction

Bipolar disorder (BD) is a common and complex psychiatric disorder with a lifetime prevalence of approximately 1% [1]. It is characterized by mood dysregulation, encompassing episodes of mania, hypomania, and depression. The disorder has strong genetic components, with an estimated heritability of approximately 80% [2], alongside contributions from various environmental factors. BD exhibits clinical and genetic heterogeneity and is often associated with substantial cognitive, emotional, and functional impairments, affecting individuals' quality of life [3, 4]. Despite advancements in pharmacological and psychotherapeutic interventions, BD remains a leading cause of disability worldwide, as quantified by years lived with disability in the Global Burden of Disease Study [5]. Therefore, further understanding the biological mechanisms underlying BD is crucial for developing more effective diagnostic tools and treatment strategies.

One promising area of research focuses on epigenetics, which examines stable changes in gene expression that occur without alterations to the DNA sequence itself. Among epigenetic mechanisms, DNA methylation has received particular attention in psychiatric research because of its flexibility and environmental sensitivity. Abnormal DNA methylation patterns have been implicated in several psychiatric disorders, such as schizophrenia, depression, and anxiety disorders [6, 7, 8, 9], suggesting that epigenetic modifications may play a critical role in the pathophysiology of BD. The ability to measure DNA methylation in accessible tissues, such as living peripheral blood, provides an opportunity to identify biomarkers for psychiatric disorders. In addition, postmortem brain studies offer perspectives on disorder‐specific changes in brain tissues directly relevant to psychiatric disorders, such as the hippocampus, a region implicated in mood regulation and cognitive processes [10, 11].

The hippocampus is of particular interest in BD because of its involvement in emotional regulation, memory consolidation, and the stress response [12]. Neuroimaging studies have consistently demonstrated decreased hippocampal volumes in patients with BD [13, 14], whereas molecular studies have revealed aberrant gene expression patterns in the hippocampal region in patients with BD [15]. Despite these findings, the extent to which epigenetic changes in peripheral tissues reflect those in the brain remains unclear. Trans‐tissue comparisons, which explore the relationship between epigenetic changes in peripheral blood and brain tissues, are essential for understanding the systemic and localized contributions of DNA methylation to the pathophysiology of psychiatric disorders, including BD.

Epigenome‐wide association studies (EWASs) have emerged as an effective approach for identifying differentially methylated positions (DMPs) associated with the risk of disorders and their related intermediate phenotypes. By systematically exploring DNA methylation across the genome, EWASs can uncover loci that may serve as biomarkers or therapeutic targets [16, 17]. Moreover, methylation risk scores (MRSs), which aggregate the effects of multiple DMPs across the genome, provide a quantitative measure of an individual's epigenetic risk for a given disorder or their related intermediate phenotypes [18, 19, 20]. Although MRSs have been applied to specific psychiatric disorders, such as schizophrenia and anxiety disorders [18, 19, 21], their application to BD remains limited.

Previous studies have focused primarily on DNA methylation in either living peripheral blood or postmortem brain tissues [7, 8, 9, 22, 23, 24, 25], often without integrating findings across tissues. Living peripheral blood studies offer the advantages of accessibility and the potential for longitudinal monitoring but are confounded by cell‐type heterogeneity. Moreover, because psychiatric disorders are fundamentally brain disorders, peripheral blood may not fully reflect the underlying pathophysiological changes occurring in the central nervous system (CNS). This raises concerns about the extent to which findings from blood‐based studies are directly relevant to the brain. Conversely, postmortem brain studies provide direct perspectives on CNS pathology but are limited by sample availability and the potential for postmortem artifacts such as cause of death. Integrating data from both blood and brain tissues allows for a more comprehensive understanding of the systemic and tissue‐specific contributions of epigenetic changes to BD. Furthermore, distinguishing disorder‐specific epigenetic alterations from confounding factors, such as smoking, body mass index (BMI), and suicide, is essential for interpreting the biological relevance of these findings. Combining epigenetic data across tissues has the potential to enhance our understanding of the epigenetic architecture of BD and may provide a basis for novel diagnostic and therapeutic approaches.

We hypothesized that MRSs derived from EWASs for BD risk in living peripheral blood and postmortem hippocampal tissues would be associated with BD diagnosis across tissue types. For example, MRSs for BD in postmortem hippocampal tissue samples differ between patients with BD and unaffected controls in living blood samples. To address the limitations of previous studies, which often focused on either living peripheral blood or postmortem brain tissues in isolation without integrating findings across tissue types, the present study performed trans‐tissue MRS analyses using EWASs for BD from two independent datasets: peripheral blood samples from living individuals (*n* = 40) and postmortem hippocampal tissues (*n* = 63). Specifically, we investigated whether MRSs for BD risk in peripheral blood obtained from living donors and postmortem hippocampal tissues are associated with BD diagnostic status across tissues. Additionally, we explored the extent to which the associations between MRSs and BD diagnosis are influenced by genome‐wide association study (GWAS) loci related to BD [26] and by health‐related confounding factors such as smoking and BMI.

## 2 Materials and Methods

### 2.1 BD Case–Control Study Using Peripheral Blood Samples From Living Donors and Postmortem Hippocampal Tissues

Two distinct sets of raw intensity data (IDAT) files were analyzed: one from peripheral blood samples of patients living with BD and unaffected controls (GSE68777_RAW) [24] and another from postmortem hippocampal tissue samples of patients with BD and nonpsychiatric controls (GSE129428_RAW) [25]. These datasets were sourced from the Gene Expression Omnibus (GEO, https://www.ncbi.nlm.nih.gov/geo/).

The living peripheral blood case–control group included 20 patients with BD and 20 unaffected controls (*n* = 40, Table 1). The postmortem hippocampal case–control group included 32 patients with BD type I (*n* = 31 after quality control for methylation) and 32 nonpsychiatric controls (*n* = 63, Table 2). The recruitment and diagnostic procedures for these studies have been detailed previously [24, 25]. Blood samples were obtained from BD patients experiencing acute manic or hypomanic episodes recruited from Sheppard Pratt Hospital in Baltimore, MD, USA. Diagnoses were made based on the following criteria: BD I, single manic episode; BD I, most recent episode manic; BD I, most recent episode mixed; BD type II, most recent episode hypomanic; or schizoaffective disorder, BD type (manic, hypomanic, or mixed state). These diagnoses followed the *Diagnostic and Statistical Manual of Mental Disorders* (DSM‐IV) based on the Structured Clinical Interview for DSM‐IV (SCID) and available medical records. Blood control individuals without the presence of a current or past psychiatric disorder based on the SCID‐Nonpatient Edition were recruited from posted announcements at local health care facilities and universities in the same geographic area as the setting where the patients were recruited. Individuals in both case–control groups met the following additional criteria: age 18–50 years (the controls were aged 20–60); absence of any history of intravenous substance abuse, intellectual disability, HIV infection, serious medical disorder that would affect cognitive functioning; or a primary diagnosis of alcohol or substance use disorder.

**TABLE 1:** Demographic characteristics of living peripheral blood tissue from unaffected controls and patients with bipolar disorder.

Variable	Unaffected controls	Bipolar disorder	*p* values (*z* or *χ* ^2^)
	(*n* = 20)	(*n* = 20)	
Predicted age (years)	25.8 ± 7.6	36.1 ± 15.3	0.13 (1.5)
Sex (male/female)	6/14	8/12	0.51 (0.4)^a^
Race (Caucasian/Other)	20/0	20/0	—
B cells	0.111 ± 0.044	0.101 ± 0.056	0.53 (−0.6)
CD4^+^ T cells	0.318 ± 0.111	0.357 ± 0.129	0.25 (1.2)
CD8^+^ T cells	0.166 ± 0.057	0.143 ± 0.087	0.30 (−1.1)
Monocytes	0.069 ± 0.039	0.099 ± 0.052	**0.049 (2.0)**
Neutrophils	0.184 ± 0.160	0.164 ± 0.260	0.16 (−1.4)
Natural killer cells	0.140 ± 0.099	0.129 ± 0.070	0.76 (−0.3)

*Note:* Age and six cell types (B cells, CD4^+^ T cells, CD8^+^ T cells, monocytes, neutrophils, and natural killer cells) in the peripheral blood were estimated using the Illumina Human Methylation Microarray. Mean ± SDs are shown. *p* values < 0.05 are shown in boldface.

^a^ *χ* ^2^ test.

**TABLE 2:** Demographic characteristics of nonpsychiatric controls and patients with bipolar disorder in postmortem hippocampal tissue.

Variable	Nonpsychiatric controls	Bipolar disorder	*p* values (*z* or *χ* ^2^)
	(*n* = 32)	(*n* = 31)	
Age (years)	48.3 ± 10.6	47.0 ± 10.4	0.57 (−0.6)
Sex (male/female)	19/13	15/16	0.38 (0.8)^a^
Race (Caucasian/Other)	16/16	26/5	**4.36 × 10** ^**−3**^ **(8.1)** ^a^
Postmortem interval (hours)	30.8 ± 13.4	31.9 ± 11.1	0.48 (0.7)
Brain tissue pH	6.5 ± 0.3	6.4 ± 0.2	0.61 (−0.5)
NeuN+ cells	0.19 ± 0.07	0.15 ± 0.08	0.10 (−1.6)
NeuN− cells	0.85 ± 0.07	0.88 ± 0.08	0.11 (1.6)
Smoking (±)	4/26^b^	14/17	**6.43 × 10** ^**−3**^ **(7.4)** ^a^
BMI	33.9 ± 11.7	28.7 ± 5.9^c^	0.082 (−1.7)
Suicide (±)	0/32	19/12	**1.16 × 10** ^**−7**^ **(28.1)** ^a^

*Note:* NeuN+ and NeuN− cell types in the hippocampus were estimated using the Illumina Human Methylation Microarray. Mean ± SDs are shown. *p* values < 0.05 are shown in boldface.

Abbreviations: BMI, body mass index; NeuN−, nonneuronal; NeuN+, neuronal.

^a^ *χ* ^2^ test.

^b^ *n* = 30.

^c^ *n* = 30.

Postmortem brain tissue samples from patients with BD and nonpsychiatric controls were obtained from the Human Brain Collection Core (HBCC) at the National Institute of Mental Health (NIMH), Bethesda, MD, USA, through the Medical Examiner's Offices of Northern and Central Virginia and the District of Columbia, USA. DNA was isolated from dissected hippocampal tissue samples and transported to the University of Texas Health Science Center at Houston, TX, USA. Psychiatric diagnoses and clinical information for postmortem subjects were determined through structured interviews with next‐of‐kin as part of psychological autopsies.

Demographic and clinical data included factors such as sex and race for the blood samples (Table 1) and factors such as age at death, sex, race, BMI, smoking status, postmortem interval (PMI), tissue pH, and cause of death (presence of suicide) for the hippocampal tissue samples (Table 2). The study complied with the World Medical Association's Declaration of Helsinki and received approval from the institutional review boards of Sheppard Pratt Hospital, the National Institutes of Health, and the University of Texas Health Science Center. Written informed consent was obtained from living participants [24] or the next‐of‐kin of deceased donors [25] prior to inclusion in each study.

### 2.2 Methylation Quantification and Quality Control

Genomic DNA was extracted from living peripheral blood and postmortem hippocampal tissue samples, followed by bisulfite conversion to assess DNA methylation levels. DNA methylation in blood samples was measured using the Illumina Infinium Human Methylation 450 BeadChip array, whereas the EPIC BeadChip was used for postmortem hippocampal tissue samples (Illumina, San Diego, CA, USA).

Signal processing, including intensity measurements, background correction, and normalization of the methylation *β* values, was performed using the R package ‘*meffil*’ designed for efficient DNA methylation data analysis [27] (https://github.com/perishky/meffil/). The raw IDAT files from the Illumina methylation arrays were loaded into R software (version 4.2.3). The quality control steps excluded low‐quality probes and samples with inadequate bead numbers or detection rates based on established thresholds [18]: (i) a beadnum.samples.threshold of 0.1, indicating the maximum allowable fraction of probes with insufficient bead counts; (ii) a detectionp.samples.threshold of 0.1 for the fraction of undetected probes; (iii) a beadnum.cpgs.threshold of 0.1 to identify poorly performing probes in terms of bead count; (iv) a detectionp.cpgs.threshold of 0.1 for the fraction of undetected probes across samples; and (v) a sex.outlier.sd threshold of 10 standard deviations for identifying sex outliers.

No samples from the blood case–control group were excluded prior to quantile normalization. However, one patient from the postmortem hippocampal case–control group was excluded because of a mismatch between biological sex and predicted sex. Individual probes were removed if they failed the quality criteria. For blood samples, probes were excluded if > 10% of the samples had *p* values > 0.01 (*n* = 1552) or a bead count < 3 (*n* = 375). For postmortem hippocampal individuals, probes were excluded if > 10% of the samples had *p* values > 0.01 (*n* = 1055) or a bead count < 3 (*n* = 873).

The number of principal components (PCs) derived from control probes was optimized to adjust for technical variability for functional normalization. Functional normalization was applied after including the PCs and the slide effect, using four PCs for the blood groups and six PCs for the postmortem hippocampal groups. Probes located on sex chromosomes were excluded from further analyses. After these quality control steps, 472,003 DNA methylation sites were retained for the blood samples, and 844,374 DNA methylation sites were retained for the postmortem hippocampal samples.

To account for cellular heterogeneity, cell‐type proportions were estimated. In living blood samples, the proportions of B cells, CD4^+^ T cells, CD8^+^ T cells, monocytes, neutrophils, and natural killer cells were estimated using *meffil*'s “blood gse35069 complete” reference profile. For the postmortem hippocampal samples, the proportions of neuronal (NeuN+) and nonneuronal (NeuN−) cells were estimated using *meffil*'s “guintivano dlpfc” reference profile. For blood case–control individuals with only the mean age available, age was predicted using the *dnaMethyAge*'s “ZhangQ2019” profile on the basis of DNA methylation patterns [28]. The predicted mean ages ± standard deviations (SDs) were consistent with the chronological mean ages ± SDs for patients (36.1 ± 15.3 vs. 36.6 ± 6.6 years) and for controls (25.8 ± 7.6 vs. 26.5 ± 6.7 years) (Table 1) [24].

### 2.3 EWASs for BD in Blood and Hippocampal Tissue Samples

For the EWASs using living blood and postmortem hippocampal tissue samples, normalized *β* values were transformed into M values using the *minfi* R package [29]. DMPs linked to BD risk were identified using linear regression analysis with the *meffil* R package. In these analyses, the *M* values for each DNA methylation site were used as the dependent variable. BD case–control status was used as the independent variable. Age or predicted age, sex, proportions of blood cell types (if the proportion differed between diagnostic groups, *p* < 0.05) or NeuN+ cells for hippocampal samples; PCs of normalized *β* values significantly correlated with slide effects (*p* < 0.05); and surrogate variables were used as covariates. A strict genome‐wide significance threshold of *p* < 9.0 × 10^−8^ was applied [30], irrespective of the microarray used (450K and EPIC).

### 2.4 MRS Calculations

MRSs were calculated using genome‐wide DNA methylation sites, along with *p* values and effect sizes (coefficient *beta* values) obtained from the EWASs for BD risk in both blood and hippocampal tissue samples. To eliminate highly correlated DNA methylation sites located within a 2‐kb proximity, the CoMeBack method [31] was applied, with a Spearman correlation threshold of 0.3 for the target case–control groups [18, 19]. After the extraction of DNA methylation sites identical to those of the discovery EWASs in the target case–control individuals and the application of the CoMeBack method, only independent DNA methylation sites remained for the target individuals (hippocampus, *n* = 341,621; blood, *n* = 337,351; Table S1). Summary statistics from the EWASs were used to compute MRSs for BD risk across two tissues. MRSs were calculated from DNA methylation sites showing a nominal association with BD risk in the discovery EWASs under liberal significance thresholds: *P*_*Tcutoff*_ < 0.001, < 0.01, *P*_*T*_ < 0.05, *P*_*T*_ < 0.1, *P*_*T*_ < 0.2, *P*_*T*_ < 0.5, and *P*_*T*_ ≤ 1. For each target individual, MRSs were calculated by weighting the methylation degree of risk methylation sites by their respective effect sizes observed in the discovery EWASs. These weighted values were then summed for all DNA methylation sites within the *P*_*T*_‐defined set for each target individual. Detailed information regarding the number of DNA methylation sites at the seven *P*_*T*_ thresholds is presented in Table S1.

### 2.5 Statistical Analyses

Statistical analyses were performed using IBM SPSS Statistics 28.0 software (IBM Japan, Tokyo, Japan). Continuous variables, such as age and cell type proportions, were compared between diagnostic groups using the Mann–Whitney *U*‐test, whereas categorical variables, such as sex and suicide, were compared using Pearson's *χ* ^2^ test. Logistic regression analyses were conducted to assess the association between MRSs at various *P*_*T*_ thresholds and BD risk in blood and hippocampal tissue samples, with diagnostic status in each tissue used as a dependent variable and MRSs as independent variables. In addition, age (or predicted age), sex, blood cell proportions (if significant differences were observed between groups, *p* < 0.05) or NeuN+ cell proportions and PCs of normalized *β* values that were significantly associated with slide effects served as covariates. Nagelkerke's pseudo‐*R* ^2^ was used to estimate the proportion of variance in BD liability explained by the MRS. To isolate the variance attributable specifically to the MRS, Nagelkerke's pseudo‐*R* ^2^ value from models with covariates alone was subtracted from that of models including the MRS. This calculation provided an estimate of the incremental variance explained by the MRSs beyond covariates. A threshold of *p* < 0.05 was considered statistically significant for all the statistical tests.

## 3 Results

### 3.1 EWASs of BD Risk in Peripheral Blood Samples Obtained From Living Donors and Postmortem Hippocampal Tissues

**FIGURE 1:**
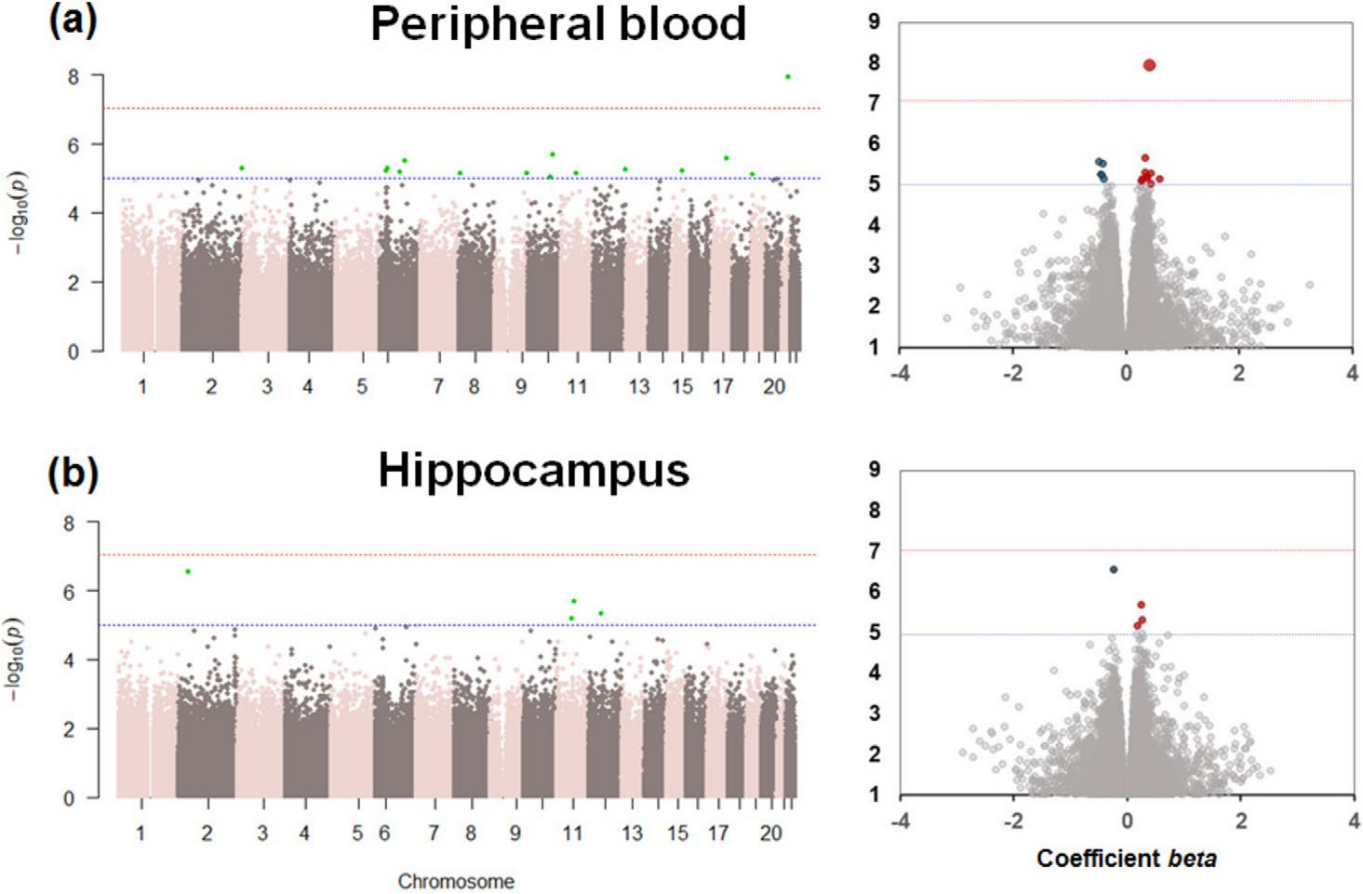
Epigenome‐wide association studies (EWASs) of the bipolar disorder (BD) risk between patients with BD and unaffected controls in (a) peripheral blood samples from living donors and (b) postmortem hippocampal tissues. Manhattan and volcano plots of differentially methylated positions (DMPs) associated with BD risk in the living peripheral blood and postmortem hippocampus, respectively, are shown. The dotted red lines in the Manhattan and volcano plots indicate a *p* value of 9.0 × 10^−8^; the dotted blue lines indicate a *p* value of 1.0 × 10^−5^. The red and blue circles in the volcano plots indicate hyper‐ and hypomethylated DMPs associated with BD risk, respectively.

Two preliminary EWASs were performed to evaluate BD risk by comparing patients with BD to unaffected controls. One EWAS used living peripheral blood samples (Figure 1a), and the other used postmortem hippocampal tissues (Figure 1b). In the blood EWAS, a genome‐wide significant DMP was identified (cg23322242, *beta* = 0.44, *p* = 1.19 × 10^−8^; Figure 1a) that was located in the intergenic region between the claudin 14 (*CLDN14*) and chromatin assembly factor 1 subunit B (*CHAF1B*) genes on chromosome 21. This DMP exhibited hypermethylation in patients with BD compared with unaffected controls. In contrast, the EWAS conducted on postmortem hippocampal tissues did not identify any genome‐wide significant DMPs (Figure 1b, *p* > 9.0 × 10^−8^).

### 3.2 Trans‐Tissue Effects of MRSs for BD Risk Across Living Peripheral Blood and Postmortem Hippocampal Tissues

**FIGURE 2:**
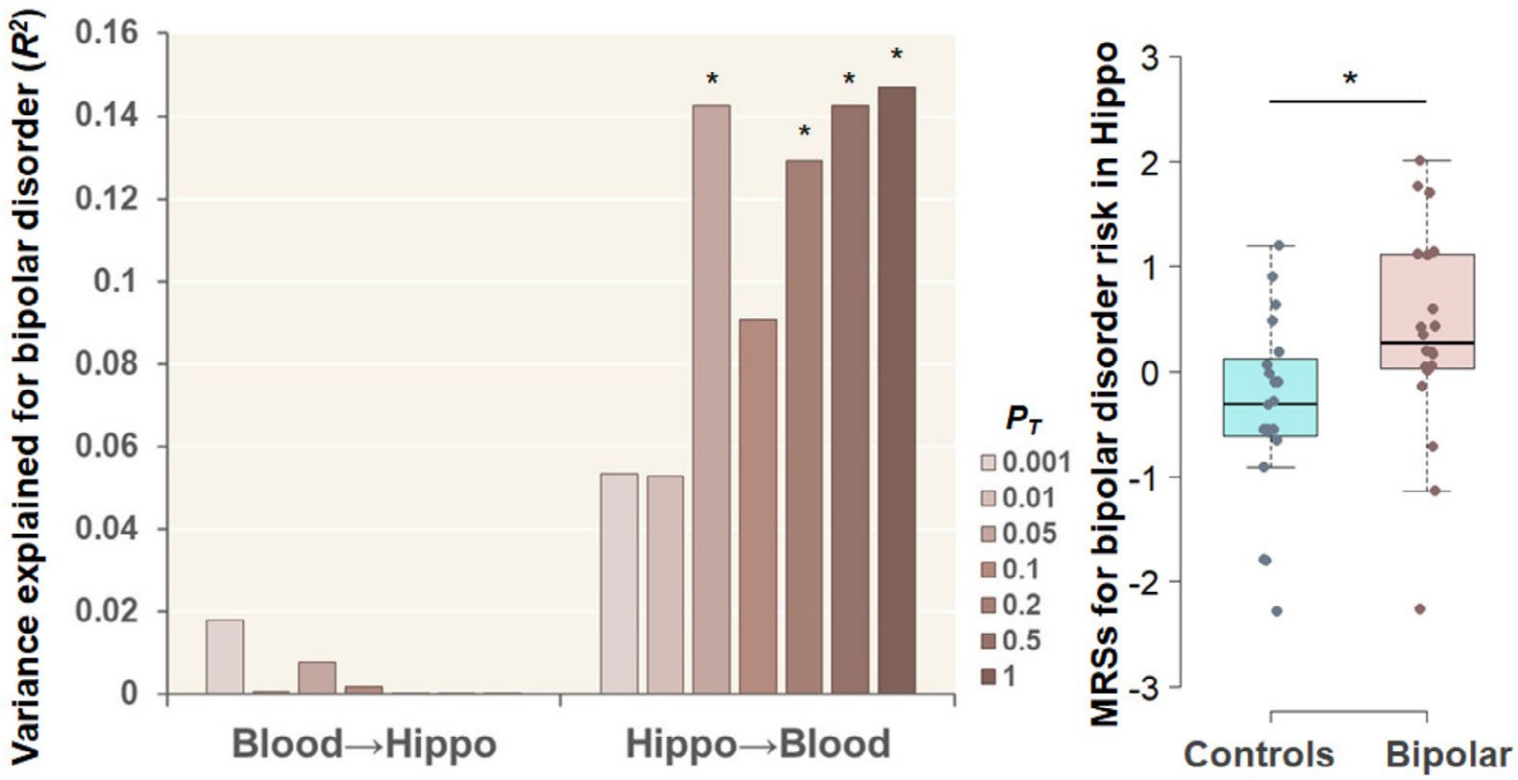
Trans‐tissue effects of methylation risk scores (MRSs) for bipolar disorder (BD) risk in peripheral blood samples obtained from living donors and the postmortem hippocampus (Hippo) at different *P*_*T*_ levels on BD diagnosis across blood and the Hippo. For example, Hippo→Blood means that EWAS of BD risk in Hippo and BD case–control individuals in blood were used as discovery EWAS and target samples, respectively. Box plot indicating the MRSs for BD risk in the Hippo region at *P*_*T*_ ≤ 1 in unaffected controls and patients with BD in the blood. The MRSs corrected for covariates were *z*‐standardized (*p < 0.05).

We investigated whether MRSs derived from EWASs of BD risk in peripheral blood samples obtained from living donors and postmortem hippocampal tissues were associated with BD diagnosis across tissues at varying *P*_*T*_ thresholds (Figure 2). The MRSs for BD risk derived from postmortem hippocampal tissues were significantly greater in patients with BD than in unaffected controls using peripheral blood samples obtained from living donors (Figure 2), with a maximum at *P*_*T*_ ≤ 1 (*R* ^2^ = 0.147, *p* = 0.026). The incremental Nagelkerke's pseudo‐*R* ^2^ showed that hippocampus‐derived MRSs explained additional variance in BD diagnosis beyond covariates. Conversely, no significant differences in MRSs for BD risk derived from living peripheral blood were detected in the postmortem hippocampus between patients with BD and unaffected controls (Figure 2, *p* > 0.05).

### 3.3 Effects of MRSs on BD Risk Derived From CpG Sites Excluding or Including GWAS‐Identified GWS Loci

**FIGURE 3:**
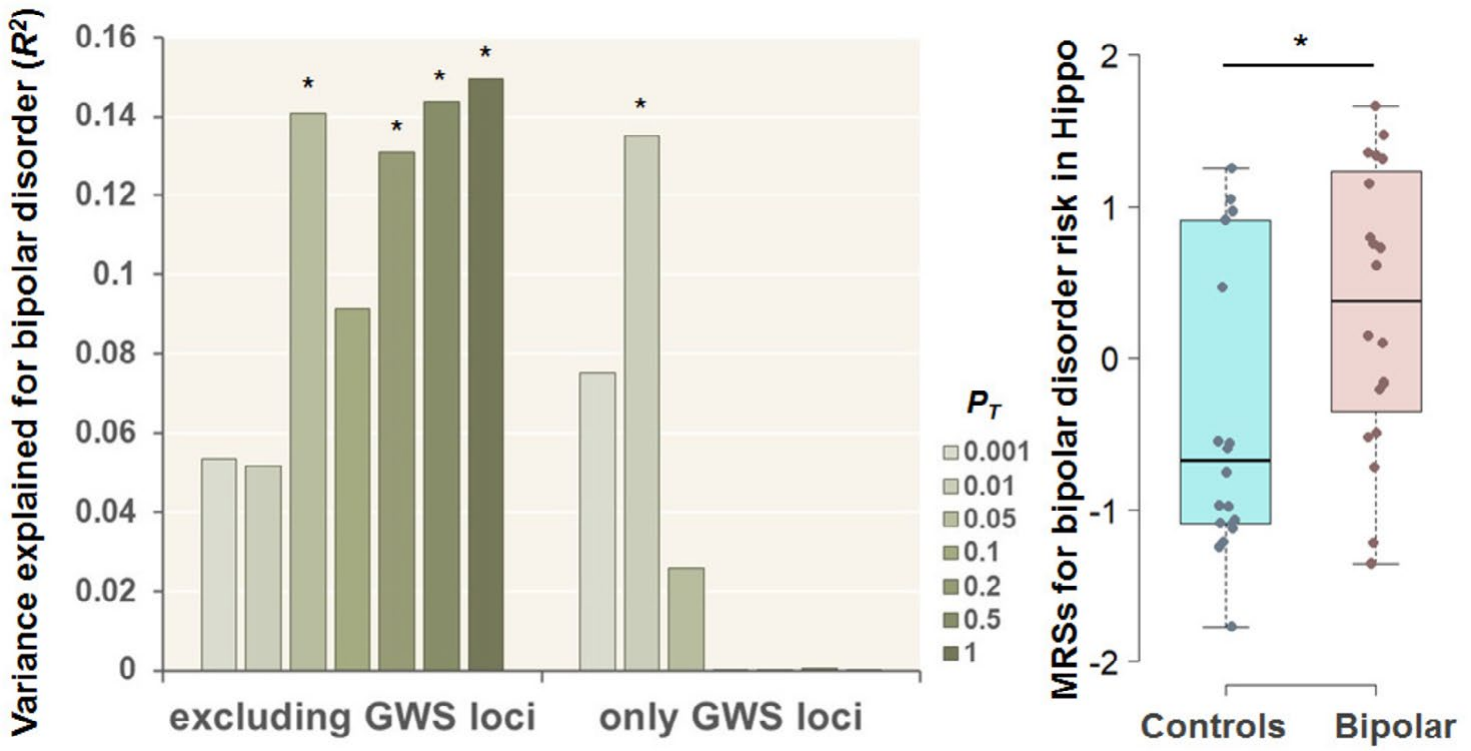
Effects of MRSs for bipolar disorder (BD) risk in the Hippo region at different *P*_*T*_ levels, derived from CpG sites excluding or including CpG sites around only GWS loci (lead SNP ±100 kb) detected by the latest and largest‐scale GWAS of BD [26], on BD diagnosis in blood. Box plot indicating the MRSs for BD risk in the postmortem hippocampus at *P*_*T*_ < 0.01, derived from CpG sites around only GWS loci, in the blood of unaffected controls and patients with BD. GWS, genome‐wide significant; SNP, single‐nucleotide polymorphism. The MRSs corrected for covariates were *z*‐standardized (*p < 0.05).

The latest large‐scale GWAS of BD by the Psychiatric Genomics Consortium wave 3 (PGC3) identified 64 genome‐wide significant (GWS) loci associated with BD in 41,917 BD patients and 371,549 controls [26]. To determine whether CpG sites near these GWS loci influenced the observed differences in postmortem hippocampal MRSs for BD risk between diagnostic groups in living peripheral blood, we additionally examined postmortem hippocampal MRSs derived from CpG sites either excluding or including only those near GWS loci (lead SNP ±100 kb, Table S2) (Figure 3). Even when CpG sites near GWS loci were excluded, postmortem hippocampal MRSs for BD risk were significantly greater in patients with BD than in unaffected controls in living peripheral blood, with a maximum at *P*_*T*_ ≤ 1 (Figure 3, *R* ^2^ = 0.150, *p* = 0.025). Moreover, MRSs derived exclusively from only CpG sites (*n* = 49, Table S2) near GWS loci at *P*_*T*_ < 0.01 were also significantly greater in patients with BD than in unaffected controls in living peripheral blood (Figure 3, *R* ^2^ = 0.135, *p* = 0.030). However, no significant differences were observed at other *P*_*T*_ thresholds (Figure 3, *p* > 0.05).

### 3.4 Effects of MRSs for Health‐Related Confounding Factors in the Postmortem Hippocampus on BD Diagnosis in Living Peripheral Blood

**FIGURE 4:**
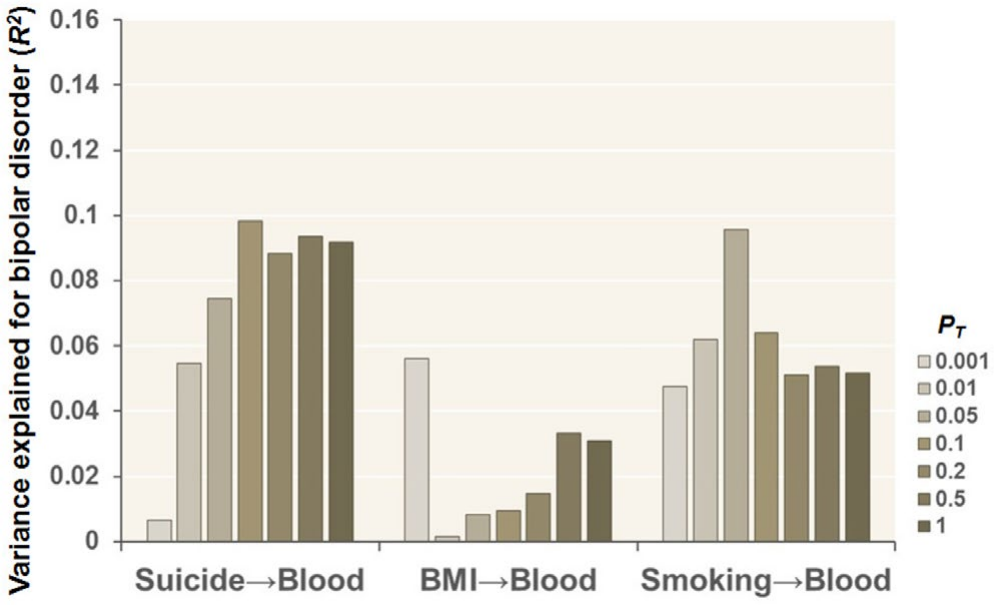
Effects of MRSs for health‐related confounding factors, such as suicide, BMI, and smoking, in the postmortem hippocampus of bipolar disorder (BD) case–control individuals at different *P*_*T*_ levels on BD diagnosis using peripheral blood samples collected from living donors.

We further explored whether MRSs based on EWASs of health‐related confounding factors, such as suicide, BMI, and smoking, in the postmortem hippocampus of BD case–control individuals differed between patients with BD and unaffected controls in living peripheral blood (Figure 4). No significant differences in MRSs related to suicide, BMI, or smoking in the postmortem hippocampus were detected between these groups in the living peripheral blood (Figure 4, *p* > 0.05), indicating that BD diagnostic status, compared with health‐related confounding factors such as suicide, BMI, and smoking in the postmortem hippocampus, may influence BD diagnostic status using peripheral blood samples obtained from living donors.

## 4 Discussion

This is the first study to investigate whether whole‐genome methylation profiles derived from EWASs of BD risk are associated with independent BD diagnostic status, particularly across living peripheral blood and postmortem hippocampal tissue samples. Our findings revealed that MRSs for BD risk, calculated from postmortem hippocampal tissue samples, were significantly associated with BD diagnosis in living peripheral blood samples. Conversely, MRSs derived from blood samples were not significantly associated with BD diagnosis in postmortem hippocampal tissue samples. Furthermore, the observed associations between MRSs in postmortem hippocampal tissue samples and BD diagnosis were not influenced by CpG sites located near GWS loci associated with BD or by health‐related confounding factors, including smoking, BMI, or suicide. These findings suggest that whole‐genome methylation profiles derived from the CNS may play a broader and more sensitive role in the pathogenesis of BD, as reflected in living peripheral blood samples.

The differential associations of postmortem hippocampus‐derived and blood‐derived MRSs with BD diagnosis may reflect the distinct biological processes captured by these tissues. Postmortem hippocampus‐derived MRSs may predominantly represent CNS‐specific epigenetic modifications associated with neuronal activity and mood regulation [32], as the hippocampus plays a major role in emotional and cognitive processes relevant to BD pathophysiology [26]. In contrast, blood‐derived MRSs may predominantly reflect peripheral inflammation and immune responses. Although such systemic inflammatory processes are associated with BD [33, 34], they are not disorder‐specific and can be influenced by external factors such as stress, smoking, or physical health conditions. Therefore, hippocampal epigenetic patterns may offer greater sensitivity to the underlying neurobiological mechanisms of BD, whereas blood‐derived MRSs might primarily indicate broader systemic effects. Additionally, brain methylation profiles are more likely to capture long‐term neurobiological changes [32], whereas blood methylation may reflect acute environmental or physiological influences [35], thus contributing to the diagnostic relevance of postmortem hippocampus‐derived MRSs.

We have previously shown that MRSs derived from the frontal cortex (*n* = 45) and superior temporal gyrus (*n* = 86), as well as blood‐derived MRSs for schizophrenia (*n* = 96 and *n* = 1714), are associated with schizophrenia diagnostic status across tissue samples [18]. Additionally, blood‐derived MRSs for social anxiety disorder (*n* = 143) are associated with the risk of panic disorder in blood samples (*n* = 263) [19]. In this study, however, our sample sizes for postmortem hippocampus‐derived MRSs (*n* = 63) and especially blood‐derived MRSs (*n* = 40) for BD patients were relatively smaller than those used in previous studies. Furthermore, the sample size of the discovery EWAS used to derive MRSs is critical, as smaller discovery EWAS datasets limit the ability to capture robust and generalizable DNA methylation signatures associated with disorder risk. Despite this limitation, we demonstrated significant trans‐tissue effects of postmortem hippocampus‐derived MRSs for the diagnosis of BD in living peripheral blood, highlighting the relevance of CNS‐specific epigenetic modifications in the pathophysiology of BD. Importantly, although blood‐derived MRSs did not show significant associations in this study, it is plausible that larger sample sizes could improve the statistical power to detect such associations and potentially minimize the observed differences between tissues. Blood‐derived MRSs offer a practical and noninvasive approach for identifying biomarkers for psychiatric disorders, making them highly promising for clinical application. Future studies should focus on increasing sample sizes and integrating multitissue analyses to validate these findings and further investigate the diagnostic utility of blood‐derived MRSs.

The observed associations between postmortem hippocampus‐derived MRSs and BD diagnostic status in blood samples were not influenced primarily by CpG sites near the 64 GWS loci associated with BD [26]. In contrast, postmortem hippocampus‐derived MRSs based exclusively on CpG sites (*n* = 49) near GWS loci at *P*_*T*_ < 0.01 were also associated with BD diagnosis in blood samples (Figure 3). These findings suggest that although GWS loci and MRSs are generally considered to be independent in terms of their contributions to the risk of psychiatric disorders [7, 18, 36], specific GWS loci may influence some methylation patterns that are relevant to the pathophysiology of BD. For example, some GWS loci identified through GWAS might modulate nearby CpG sites through genetic effects such as methylation quantitative trait loci (meQTLs), potentially impacting the expression of genes involved in neural signaling, synaptic plasticity, or the stress response. This highlights the possibility that GWS loci not only confer genetic risk but also may interact with epigenetic processes to modulate these biological functions.

No associations between smoking‐, BMI‐, or suicide‐derived MRSs in postmortem hippocampal tissue samples and BD diagnosis in blood samples were observed. This lack of association suggests that although these factors are known to influence DNA methylation patterns and may contribute to the broader epigenetic profile, their specific contributions do not appear to play a direct role in the cross‐tissue diagnostic status of BD. Smoking and BMI are well‐demonstrated lifestyle‐related factors that broadly affect methylation across various tissues, particularly in peripheral samples [37, 38]; however, their impact on brain‐derived methylation profiles may be more localized or independent of the disorder‐specific changes associated with BD. Similarly, suicide‐related methylation changes, which have been linked to stress‐related gene regulation and neural plasticity [39, 40], might reflect end‐stage or acute processes that are not sufficiently represented in the peripheral blood profiles of living individuals. These findings highlight the importance of distinguishing between disorder‐specific epigenetic changes and those resulting from lifestyle or environmental factors.

There are limitations to the interpretation of our findings. As mentioned above, the number of living peripheral blood samples was relatively small. Consequently, we cannot definitively exclude the effect of blood‐derived MRSs on BD diagnosis in postmortem hippocampal tissue samples. The lack of observed associations may become significant in studies performed using an increased number of living peripheral blood samples. In the blood samples, health‐related information such as smoking status and BMI was not available, which might have affected our findings. Additionally, the diagnosis of patients with BD differed between studies. Specifically, BD I was the focus of the hippocampal study, whereas a mix of BD subtypes was included in the blood study. Moreover, because the discovery datasets were generated using different microarray platforms (450K and EPIC), we calculated MRSs separately within each dataset. Although integration is technically possible by restricting analyses to common CpG sites, no independent target BD samples are currently available to validate such integrated MRSs. Furthermore, hippocampal alterations are not specific to BD but are also observed in other psychiatric disorders, which may limit the disorder specificity of our findings. In addition, BD‐specific MRSs derived from large‐scale EWASs are currently not available for external validation, and future large‐scale, multi‐tissue EWASs are needed. We evaluated the contribution of MRSs using Nagelkerke's pseudo‐*R* ^2^, which showed that MRSs explained additional variance in BD diagnosis beyond covariates. However, the absence of paired blood–hippocampal samples, technical replicates, and the limited sample sizes restricted our ability to perform stable variance decomposition. Future studies with larger, paired multi‐tissue datasets are needed to disentangle the respective contributions of BD diagnosis, data source, and within‐subject error.

## 5 Conclusion

We highlight the diagnostic relevance of postmortem hippocampus‐derived MRSs in living peripheral blood samples and propose that CNS‐derived epigenetic profiles hold potential as sensitive biomarkers for BD. Although most CpG sites near GWS loci did not make substantial contributions, we highlight that MRSs derived from a subset of these sites were associated with diagnosis, suggesting a possible interaction between genetic and epigenetic factors. In contrast, we found no associations between smoking‐, BMI‐, and suicide‐derived MRSs and BD diagnosis, reflecting the distinct features of the observed effects. Although blood‐derived MRSs showed no significant associations with postmortem hippocampal tissue, larger sample sizes may reveal their diagnostic utility. These findings emphasize the necessity of future studies with expanded datasets and multitissue analyses to better clarify the complex relationships among genetic, epigenetic, and environmental factors in BD.

## Funding

This work was supported by the Grants‐in‐Aid for Scientific Research (C) (22K07614, 25K10832, and 25K10808) from the Japan Society for the Promotion of Science (JSPS); the AMED under grant numbers JP21uk1024002, JP22dk0307112 and JP23dk0307103; a grant from the Takeda Science Foundation (N/A); and a grant from the Mochida Memorial Foundation for Medical and Pharmaceutical Research (N/A).

## Ethics Statement

The study complied with the World Medical Association's Declaration of Helsinki and received approval from the institutional review boards of the Sheppard Pratt Hospital, the National Institutes of Health, and the University of Texas Health Science Center.

## Consent

Written informed consent was obtained from living participants or the next‐of‐kin of deceased donors prior to inclusion in each study.

## Conflicts of Interest

The authors declare no conflicts of interest.

## Supporting information


**Table S1:** Number of CpG sites at the seven *P*_*T*_ thresholds used in the MRS calculations for the target case–control blood and hippocampus samples.
**Table S2:** Number of CpG sites at the seven *P*_*T*_ thresholds used in MRS calculations for CpG sites excluding or including only GWS loci in the target case–control blood samples.

## Data Availability

The datasets generated and/or analyzed during the current study are available from the corresponding author upon reasonable request. The raw IDAT data are publicly available from the Gene Expression Omnibus (GEO, https://www.ncbi.nlm.nih.gov/geo/).
