# Supplementary material for: Trans‐Tissue Effects of Hippocampus‐ and Blood‐Derived DNA Methylation Risk Scores on Bipolar Disorder Diagnosis
Source: Bipolar Disord. 2026 Jan 24;28(1):e70078. doi: 10.1111/bdi.70078 (PMC12831226; doi:10.1111/bdi.70078)
Supplement: Supplementary file 1 — Table S1: Number of CpG sites at the seven P T thresholds used in the MRS calculations for the target case–control blood and hippocampus samples. Table S2: Number of CpG sites at the seven P T thresholds used in MRS calculations for CpG sites excluding or including only GWS loci in the target case–control blood samples. [file BDI-28-0-s001.docx]

**Supplementary Information**

**Supplementary Table 1.** Number of CpG sites at the seven *P_T_* thresholds used in the MRS calculations for the target case‒control blood and hippocampus samples.

| Discovery case‒control EWASs | Blood |  | Hippocampus |
| --- | --- | --- | --- |
| Illumina Methylation array | 450K |  | EPIC |
| Target case‒control sample | Hippocampus |  | Blood |
| Illumina Methylation array | EPIC |  | 450K |
| *P_T_*<0.001 | 1,144 |  | 807 |
| *P_T_*<0.01 | 8,443 |  | 5,931 |
| *P_T_*<0.05 | 32,940 |  | 25,500 |
| *P_T_*<0.1 | 57,869 |  | 47,539 |
| *P_T_*<0.2 | 100,677 |  | 87,868 |
| *P_T_*<0.5 | 202,816 |  | 191,421 |
| *P_T_*≤1 | 341,621 |  | 337,351 |

MRS, methylation risk score; EWASs, epigenome-wide association studies.

**Supplementary Table 2.** Number of CpG sites at the seven *P_T_* thresholds used in MRS calculations for CpG sites excluding or including only GWS loci in the target case‒control blood samples.

| CpG sites | Excluding GWS loci |  | Including only GWS loci |
| --- | --- | --- | --- |
| *P_T_*<0.001 | 805 |  | 2 |
| *P_T_*<0.01 | 5,882 |  | 49 |
| *P_T_*<0.05 | 25,313 |  | 189 |
| *P_T_*<0.1 | 47,160 |  | 382 |
| *P_T_*<0.2 | 87,109 |  | 773 |
| *P_T_*<0.5 | 189,735 |  | 1,748 |
| *P_T_*≤1 | 334,305 |  | 3,221 |

MRS, methylation risk score; GWS, genome-wide significance. Because the CpG sites were grouped using CoMeBack after the CpG sites were divided into those excluding or including only GWS loci, the total number of CpG sites at each *P_T_* (Supplementary Table 2) was greater than that in the target case‒control blood samples, as shown in Supplementary Table 1.
